# Two-Dimensional Projected-Momentum Covariance Mapping for Coulomb Explosion Imaging

**DOI:** 10.1021/acs.jpca.4c01084

**Published:** 2024-04-12

**Authors:** Joseph W. McManus, Felix Allum, Josh Featherstone, Chow-Shing Lam, Mark Brouard

**Affiliations:** Chemistry Research Laboratory, Department of Chemistry, University of Oxford, Oxford OX1 3TA, U.K.

## Abstract

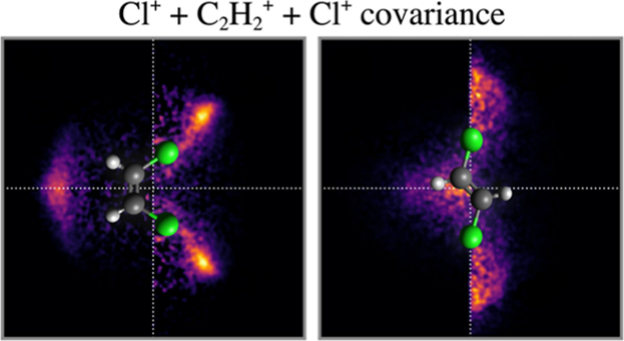

We introduce projected-momentum covariance mapping, an extension of recoil-frame covariance mapping for 2D ion imaging studies. By considering the two-dimensional projection of the ion momenta as recorded by the detector, one opens the door to a complex suite of analysis tools adapted from three-dimensional momentum imaging studies. This includes the use of different frames of reference to unravel the dynamics of fragmentation and the application of fragment momentum constraints to isolate specific fragmentation channels. The technique is demonstrated on data from a two-dimensional ion imaging study of the Coulomb explosion of the *cis* and *trans* isomers of 1,2-dichloroethene, following strong-field ionization by an intense near-infrared femtosecond laser pulse. Classical simulations are used to guide the interpretation of projected-momentum covariance maps. The results offer a detailed insight into the distinct Coulomb explosion dynamics for this pair of isomers and lay the groundwork for future time-resolved studies of photoisomerization dynamics in this molecular system.

## Introduction

Coulomb explosion imaging (CEI) has become a widely adopted technique for probing molecular structure on an ultrafast time scale.^1,2^ This is achieved by inducing Coulomb explosion of a target molecule, the process whereby multiple electrons are rapidly removed to form a molecular polycation, which subsequently “explodes” into many fragment ions. The relative momenta of the resulting fragments are measured from which information about the nuclear structure at the instant of Coulomb explosion can be inferred. CEI has been extensively used to determine static nuclear structure, such as distinguishing structural^3−6^ and chiral^7−9^ isomers, and identifying different conformations of molecular clusters.^10^ It is of particular interest when combined with pump–probe spectroscopy to explore time-evolving nuclear structure. Such time-resolved CEI (TR-CEI) studies have probed photoinduced molecular dynamics such as vibration,^11,12^ dissociation,^13−16^ and isomerization.^17,18^

In this work, we focus on how the fragment momenta are measured and the implication that it has on the structural information that can be extracted. The main method of doing so is ion imaging^19^ wherein each fragment ion is accelerated onto a position sensitive detector. The in-plane components of each ion’s momentum can then be calculated directly from its impact position, while the third out-of-plane component must be reconstructed from its arrival time. Recording the ion time-of-flight (TOF) with sufficient resolution to perform this final step can be a challenge, so it is common to omit the reconstruction of the third momentum component, in which case only a two-dimensional (2D) projection of the ion momentum onto the detector is recorded.^20,21^ In the current work, this is referred to as 2D projected-momentum imaging, in contrast to three-dimensional (3D) momentum imaging,^7,22−29^ where all three components are measured with comparable precision.

For cylindrically symmetric velocity distributions, it is a straightforward process to retrieve the 3D fragment momentum distribution from its 2D projection using an inverse Abel transform.^30^ However, in CEI, it is the relative correlated momenta of multiple fragments that is of interest. Unfortunately, correlated fragment momentum maps typically do not possess the cylindrical symmetry required to perform an inverse Abel transform. Also, it is not practical to use slice imaging^31^ to measure only the central in-plane slice of the distribution. This is only a valid approach if fragment ions recoil in plane, and even then, several slices would need to be recorded per experimental cycle, one for each fragment species. This means it generally is not possible to obtain the relative 3D fragment momentum distribution in a CEI experiment that employs 2D projected-momentum imaging, and structural information must be deduced from the 2D projected-momentum correlation map.

Hansen et al. first introduced covariance mapping applied to 2D ion imaging data to examine the angular correlation between pairs of products from a Coulomb explosion.^32^ Another method of examining the fragment ion correlations for a CEI experiment which employs 2D ion imaging is “recoil-frame” covariance mapping.^20,21^ This is an intuitive approach for visualizing the relative recoil of two fragment ions, wherein the recoil vectors of a “reference” ion are rotated to lie along a common axis, and the spatial distribution of a “partner” ion on the detector is plotted relative to this direction. It is well suited for studying the Coulomb explosion between a pair of ions, such as a two-body breakup,^14,33^ or a three-body breakup involving a neutral cofragment,^33,34^ but not for studying Coulomb explosion into many ions. 3D ion imaging studies which utilize more advanced analysis techniques have been able to examine many-body fragmentation dynamics in much greater detail.^6,8,35^

The present work introduces a new analysis procedure for 2D projected-momentum imaging data that expands upon recoil-frame covariance mapping by incorporating techniques adapted from analogous 3D momentum imaging studies. This includes the use of alternative frames of reference, along with different methods of representing the data, which, combined with multiparticle correlation mapping, provide insight into the dynamics of fragmentation. Overall, this analysis methodology offers a deeper understanding of the Coulomb explosion process, producing results that are comparable with leading 3D momentum imaging experiments.

Specifically, we report on the Coulomb explosion of the *cis* and *trans* isomers of 1,2-dichloroethene (DCE) following strong-field ionization by intense near-infrared femtosecond (fs) laser pulse. This molecule was selected because it is of interest for time-resolved photochemistry studies, for example, to investigate *cis*–*trans* isomerization dynamics^36,37^—a simple form of molecular photoswitching. Several experimental studies have been conducted on the Coulomb explosion of this pair of geometric isomers^33,38−40^ plus a recent theoretical study.^41^ Ablikim et al. used the momentum correlation between the products of a three-body breakup to determine the *cis* and *trans* structures.^39^ Crane et al. have offered a thorough discussion of the various two-body breakup channels of doubly and triply charged parent ions.^33^ Notably, these experiments only investigated the Coulomb explosion of relatively low charge parent polycations (≤3). The present study expands upon the previous body of work by examining the Coulomb explosion dynamics of more highly charged parent ions in order to explore how the fragmentation dynamics change with higher parent ion charge state (*Z*).

## Methods

### Experimental Section

The experimental apparatus consists of a Ti:sapphire laser system (Spectra-Physics Solstice Ace) coupled to a velocity-map imaging^42^ (VMI) spectrometer. The setup has been described in detail in a prior publication,^43^ hence only a brief overview is provided here. A ∼10% mixture of each sample was prepared by diluting its room temperature vapor pressure in helium, up to a pressure of 3 bar. A supersonic molecular beam was produced by expanding the gaseous mixture into the vacuum chamber through a pulsed valve (Series 9 General Valve) before passing the expansion through a skimmer. The resulting collimated beam entered a set of VMI ion optics through an aperture in the rear of the repeller electrode and, in the region between the repeller and extractor electrodes, was intersected perpendicularly by the probe pulse. This is the fundamental output of the Ti:sapphire amplifier (800 nm, ∼40 fs duration), focused with a 200 mm focal length lens.

The velocity-mapping field accelerated the nascent charged particles onto a 2D position sensitive detector consisting of a pair of chevron-stacked microchannel plates coupled to a P47 phosphor screen. Each ion impact on the detector generated a flash of light which was imaged by a pixel imaging mass spectrometry (PImMS) camera^44,45^ equipped with a PImMS2 sensor—a fast timestamping camera which stores both the 2D position and time of each event with a precision of 25 ns. This allowed images of all fragment ions to be recorded simultaneously, with the timing of each event indicative of the particle’s mass-to-charge (*m*/*z*) ratio. The experiment was restricted to operating at 10 Hz due to limitations in the repetition rate at which the PImMS2 camera is able to acquire data. For each target molecule, data were acquired over ∼100,000 experimental cycles.

In previous studies using a PImMS2 camera, 3D ion imaging with modest momentum resolution along the TOF axis has been achieved by employing ion optics designed to temporally stretch the ion Newton spheres.^26,27,29^ However, in the current experiments, high ion optics voltages were required to focus fast moving fragments onto the detector, e.g., Cl^2+^. This effectively “crushed” the ion Newton spheres onto the detector, making it impossible to reconstruct the out-of-plane ion momenta with the 25 ns time-stamping precision of the PImMS2 camera.

A calibration between laser pulse energy and peak laser intensity was established based on the observed ponderomotive shift of the above-threshold ionization (ATI) peaks in the photoelectron spectra of Ar recorded at a series of pulse energies.^46^ Photoelectrons were imaged by reversing the polarity of the potentials applied to the ion optics. Data presented here were recorded with a pulse energy of 35 μJ, corresponding to a peak intensity of 4 × 10^13^ W cm^–2^. In addition, the spacing of the ATI peaks, which is equal to the photon energy, could be used to calibrate the absolute energy scale of the velocity map images. This provided a conversion between each ion’s 2D position and its in-plane momentum.

### Covariance Mapping

In order to measure the momenta of the fragments of a Coulomb explosion in correlation, the predominant method is to operate under “coincidence” conditions, where on average <1 parent ion is generated per laser shot.^3,5,7,8,11,17,18^ Multiple ions generated in a single laser shot can then be confidently assigned to breakup of the same molecule. However, this restricts the event rate at which data can be recorded and can lead to impractically long data acquisition times, especially if the repetition rate is limited, as in the current experiment. If the count rate is increased, “false” correlations between ions generated from the breakup of separate molecules begin to dominate. Fortunately, the “true” correlations can still be extracted by calculating the covariance—a statistical measure of the linear correlation between parameters across a data set of many observations (laser shots).^28,47^

The 2-fold covariance between a pair of variables is defined as
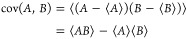
1where ⟨*i*⟩ refers to the mean of the measured quantity *i* over a series of observations. This analysis approach can extract correlated information when averaging over a large ensemble of molecules, allowing experiments to be conducted at event rates several orders of magnitude higher than coincidence techniques.^28^ Covariance mapping was first applied in the context of molecular fragmentation to study fragment TOF correlations.^47^ In the years since, the technique has been greatly expanded upon through application to a number of 2D^4,13−15,20,21,32^ and 3D^6,28,48^ CEI experiments. Several ion imaging studies have also made use of 3-fold covariance analysis^49^
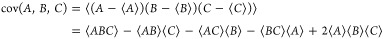
2either to assign a three-body fragmentation pathway^28,50^ or elucidate new information about the parent ion structure.^51^ Recently, procedures to extend the analysis further to four-or-more-particle correlations have also been derived^52^ and demonstrated experimentally.^53^

### Contingent Covariance

Due to the nature of covariance analysis, it relies on stable experimental conditions. Fluctuations in any experimental parameter which is correlated with the total ion signal (i.e., beam intensity) will cause the yield of all species to rise and fall as one, introducing false contributions to the calculated covariance. To account for this effect, a contingent covariance analysis^54^ was implemented which groups the raw data into 10 smaller subsets over which the fluctuating parameter is approximately constant. The covariance is then calculated separately for each subset, and the resulting set of covariance maps is averaged to give the final result. Raw data were grouped into subsets based on the total ion count per laser shot, which can serve as a proxy for any number of fluctuating parameters, eliminating the need to measure each independently.^55,56^ Aside from noise, no qualitative differences were observed between the covariance maps calculated across the 10 subsets. The total ion count per laser shot distribution for each molecule is presented in Figure S1 of the Supporting Information.

### Two-Dimensional Projected-Momentum Imaging

**Figure 1:**
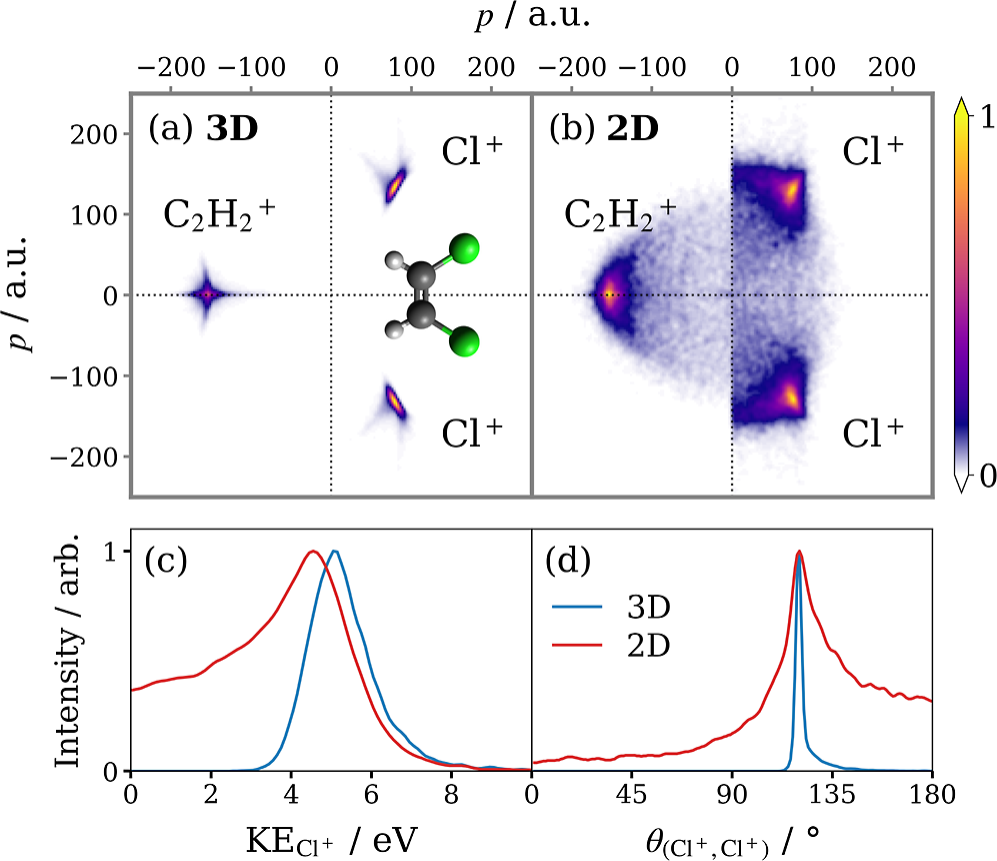
Simulated fragment momentum correlation maps for the three-body concerted dissociation of *cis*-1,2-DCE^3+^ into C_2_H_2_^+^ + 2Cl^+^, calculated using (a) the 3D fragment momenta and (b) 2D projected fragment momenta. Each panel is normalized separately. Below the (c) Cl^+^ KE and (d) (Cl^+^, Cl^+^) relative recoil angle distributions are overlaid.

Because the experimental detection system lacked sufficient time-stamping precision to reconstruct the out-of-plane component of an ion’s momentum, the experiments employed 2D projected-momentum imaging. As such, all fragment ion recoil correlations must be constructed from 2D projected-momentum information. The effect of this is demonstrated by Figure 1, in which the simulated correlated fragment momenta for the Coulomb explosion of triply charged *cis*-1,2-DCE into C_2_H_2_^+^ + 2Cl^+^ are plotted. The vertical axis in this frame of reference is defined by the relative momentum of the two Cl^+^ ions (), the result being that the horizontal axis bisects the Cl^+^ momenta. Data were simulated using a classical model of point charges interacting under Coulomb’s law,^21^ with an ensemble of starting geometries intended to approximate ground-state vibrational motion (see the Supporting Information for details).

The correlation map in panel (a) of Figure 1 has been simulated using 3D momentum information, while panel (b) displays the corresponding 2D projected-momentum correlation map. The transformation from 3D to 2D projected fragment momentum information is analogous to crushing a sphere onto a plane, which explains why the projected-momentum distribution is smeared across all angles, but only toward lower radii. Despite the modest blur, the peaks in the distribution remain distinct. From inspection of the integrated distributions in the lower panels of Figure 1, the angle of maximum intensity is unchanged, while the peak in the radial distribution is shifted down in magnitude slightly. This means fragment kinetic energies (KE) appear slightly below their true values when measured from 2D projected-momenta.

## Results and Discussion

**Figure 2:**
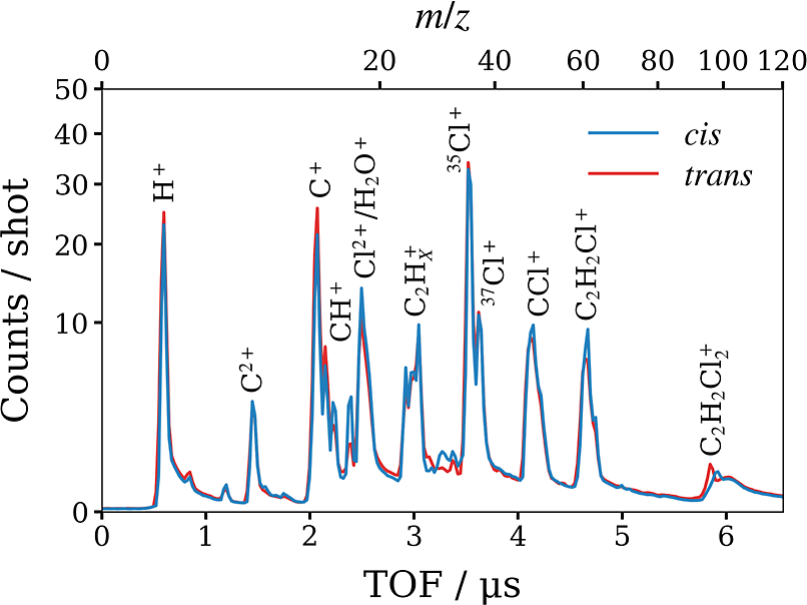
Ion TOF spectra of *cis*- and *trans*-1,2-DCE exposed to the probe pulse with an intensity of 4 × 10^13^ W cm^–2^. Unlabeled peaks are background gases. Data sets were acquired under comparable experimental conditions and are not otherwise normalized.

The ion TOF mass spectra of strong-field ionized *cis*- and *trans*-1,2-DCE recorded by the PImMS2 camera are overlaid in Figure 2. The recorded yield of parent ions is very low because, under velocity-mapping conditions, ions with near-zero transverse momentum are focused to a point on the detector, producing overlapping flashes which saturate the detector. It is also possible that the detection efficiency of this central point on the detector is somewhat diminished, but this does not meaningfully affect high KE species. A full discussion is given in the Supporting Information. The parent ion peak is broad both because there are four different unresolved isotopic masses, due to the various combinations of the two Cl isotopes, and because of the way in which the PImMS2 camera behaves when saturated, which leads to some hits bleeding into later time bins. Contributions from clusters can be ruled out based on a number of observations (see the Supporting Information).

Aside from a slightly higher yield of heavier molecular fragments (and background ions) in the *cis* spectrum, the spectra are similar, implying that the fragmentation pathways are largely isomer-independent. The host of observed ion species represents the full array of molecular fragments conceivable for the breakup of these species. This is indicative of the broad range of parent ion charge states that are generated and the assortment of different pathways via which they dissociate.

A recent ab initio trajectory study explored the *Z*-dependent Coulomb explosion dynamics for *cis*-1,2-DCE and found two distinct regimes of behavior.^41^ For “low” *Z* states, those which dissociate to yield a mixture of atomic and molecular fragments, the mapping from initial molecular geometry to final fragment momenta was complex due to the fragmentation dynamics differing so much between individual channels. By contrast, “high” *Z* states, for which dissociation results in direct and exclusive formation of atomic ions, had much simpler, Coulomb-interaction dominated mapping. In the following, we explore two fragmentation pathways, one from each regime, namely, the three-body breakup of a parent trication, and the many-body breakup of a highly charged parent ion which yields, among other atomic ions, a pair of chlorine dications.

### Three-Body Breakup

**Figure 3:**
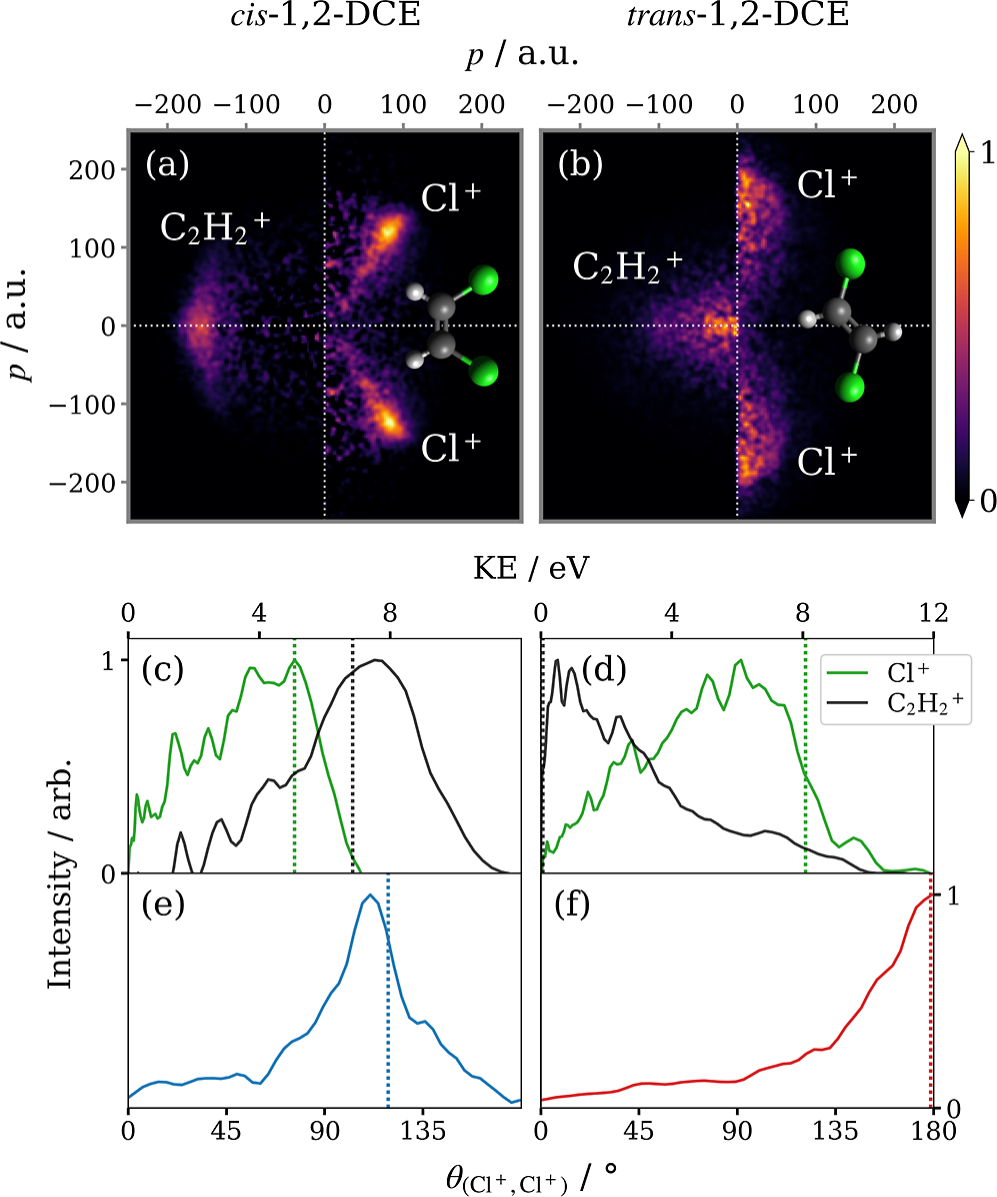
2D projected-momentum 3-fold covariance maps for the three-body breakup of (a) *cis*- and (b) *trans*-1,2-DCE^3+^ into C_2_H_2_^+^ + 2Cl^+^. Each panel is normalized separately. Below are overlaid (c,d) the fragment KE and (e,f) (Cl^+^, Cl^+^) relative recoil angle distributions. Vertical dotted lines indicate the results of a classical simulation of the Coulomb explosion of each molecule in its ground state equilibrium geometry.

The three-body breakup of triply charged 1,2-DCE into C_2_H_2_^+^ + 2Cl^+^ is the simplest, lowest total-charge Coulomb explosion channel which is expected to produce isomer-dependent signals. As such, it provides an interesting contrast to “complete” Coulomb explosion that yields exclusively atomic ions, which we examine in the next section. The correlated fragment momenta for this fragmentation channel, determined via 3-fold contingent covariance analysis, are plotted in the top panels of Figure 3. By considering the 2D projected fragment momenta, it allows only those combinations of ions which approximately fulfill momentum conservation in the plane of the detector to be selected. A constraint on the projected fragment momentum sum of ±5 a.u. greatly reduced the noise in the covariance maps and was essential to isolate the signal from this fragmentation channel. This is consistent with the width of the fragment momentum sum distribution for the two-body breakup into Cl^+^ + C_2_H_2_Cl^+^, as determined via 2-fold covariance analysis.

The covariance analysis transforms the data into the same frame of reference as described for Figure 1. This demonstrates the use of covariance analysis in a 2D ion imaging study to present fragment ion correlations in an alternative frame of reference to the conventional “recoil-frame”. In this instance, the advantage of a different frame of reference is that it allows the relative momenta of all fragment ions to be plotted together, providing an intuitive depiction of the Coulomb explosion process.

It should be noted that in the case of *trans*-1–2-DCE, if  and  are directly opposite, their difference (taken as the *y*-axis) lies parallel to . The *xy*-plane will therefore not be uniquely defined as the C_2_H_2_^+^ fragment will have zero momentum perpendicular to *y*. However, in general  and  will not be exactly opposite, and the C_2_H_2_^+^ momentum will be nonzero in the *xy* plane. In this case, the direction of the C_2_H_2_^+^ momentum is used to define the *xy*-plane.

The covariance maps in panels (a) and (b) of Figure 3 closely resemble the nuclear structures of the isomers under study. Because the Cl atoms are adjacent to one another in the *cis* isomer, both Cl^+^ recoil in the opposite direction to C_2_H_2_^+^ when the parent trication dissociates. The relative recoil angle distribution for the Cl^+^ pair is displayed in panel (e) and peaks at 111°. In the *trans* form, the Cl atoms are located on opposite sides of the C=C double bond, which causes the Cl^+^ pair to recoil approximately back-to-back, while C_2_H_2_^+^ is caged between them. The mutual repulsion on this species on average cancels out, producing low momentum C_2_H_2_^+^. Comparing this fragmentation channel for this pair of isomers is essentially comparing the repulsion of three charges arranged in a triangle (*cis*) versus a line (*trans*). From the fragment KE distributions shown in panels (c) and (d), it is clear that the linear arrangement accelerates Cl^+^ to higher velocity.

Drawn on the fragment KE and relative recoil angle distributions in Figure 3 are values calculated for the Coulomb explosion of each isomer in its ground state equilibrium geometry. These are generally in good agreement with experiment. Notably, the predicted (Cl^+^, Cl^+^) relative recoil angles are very close to the peaks in the experimental distributions. However, upon comparison of the covariance map for *cis*-1,2-DCE in Figure 3a with the simulated correlation map for the same fragmentation channel in Figure 1b, there are distinct differences. The experimental Cl^+^ momentum distribution possesses a tail, not seen in the simulated data, which trails from the point of maximum intensity down toward the origin, while the momentum distribution of C_2_H_2_^+^ is considerably broader along the vertical axis.

An analogous simulation for the Coulomb explosion of *trans*-1,2-DCE^3+^ (presented in Figure S4 of the Supporting Information) does not fully reproduce the form of the covariance map in Figure 3b, either. It predicts a distribution which is tightly confined along the vertical axis, i.e., the simulated Cl^+^ ions do not deviate significantly from back-to-back recoil, whereas in the experimental data, each fragment has a significant horizontal momentum component. Such discrepancies are indicative of more complex fragmentation dynamics. The simulation models the dissociation as a strictly concerted process, i.e., both C–Cl bonds are broken simultaneously, but that is not the case for this fragmentation channel. To examine the Coulomb explosion dynamics, we transform the data into a different frame of reference.

**Figure 4:**
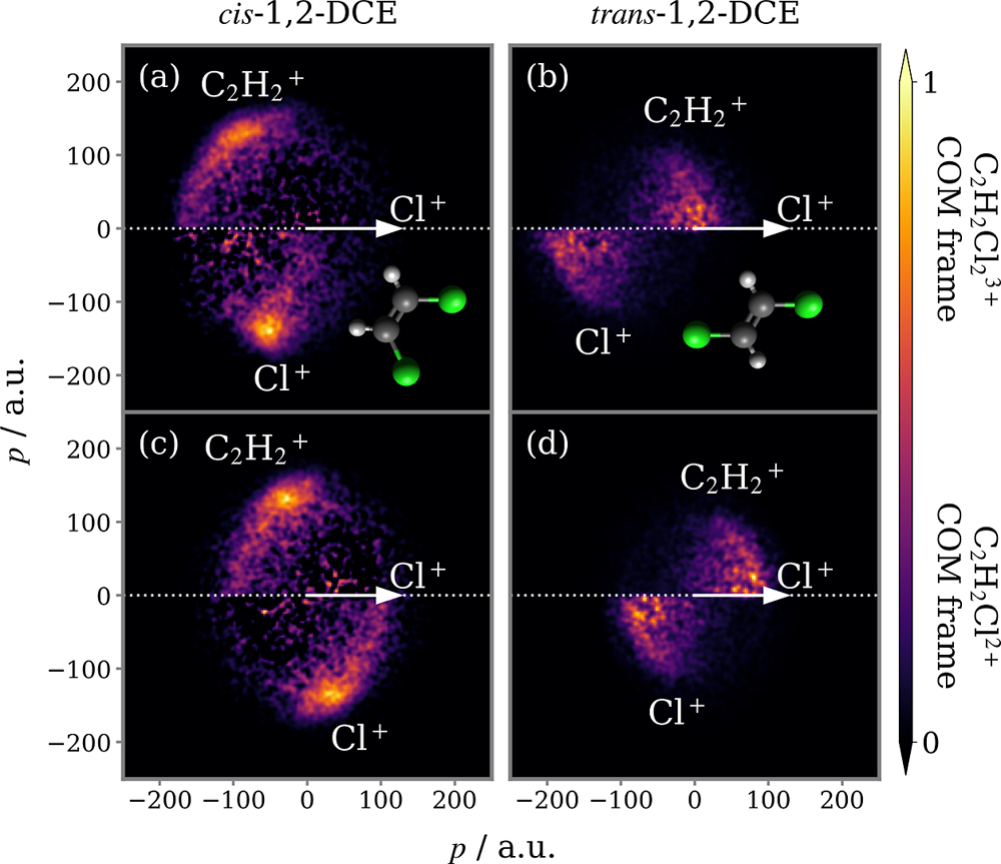
2D projected-momentum Newton diagram 3-fold covariance maps for the three-body breakup of (left column) *cis*- and (right column) *trans*-1,2-DCE^3+^ into C_2_H_2_^+^ + 2Cl^+^. Panels (a,b) display the fragment momenta in the COM frame of the parent trication. Below, in panels (c,d), fragment momenta have been transformed into the COM frame of the C_2_H_2_Cl^2+^ intermediate. Each panel is normalized separately.

One popular method for investigating the dynamics of a three-body Coulomb explosion process is to present the data as a Dalitz plot,^57^ which depicts the energy sharing among the fragments. This method has previously been adapted for the analysis of 2D projected-momentum data.^58−60^ Here, a different method is adopted. In the top panels of Figure 4, the correlated fragment momenta of Cl^+^ + C_2_H_2_^+^ + Cl^+^ have been replotted as Newton diagrams, wherein the momentum of one of the Cl^+^ ions is constrained to the positive *x*-axis, and the relative momenta of the other two fragments plotted on the top and bottom halves of the diagram.

This new representation is the same as that used in previous studies which investigated this fragmentation channel.^33,39^ The 2D projected momentum, 3-fold covariance maps show good resemblance to the 3D momentum, triple coincidence maps in these prior publications. The Cl^+^ and C_2_H_2_^+^ momenta for both isomers now appear as short arcs, curving in opposite directions. This is characteristic of a sequential three-body breakup mechanism^61,62^
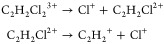
3and arises when the initial dissociation induces rotation of the intermediate dication before it further fragments, causing the momenta of the secondary products to be distributed over an angular span relative to the momentum of the primary product.

Assuming rotation of the C_2_H_2_Cl^2+^ intermediate occurs in the plane of the parent molecule, the intensity in the angular distribution of the secondary products decays exponentially from the angle for concerted breakup. The decay constant is determined by the ratio of the lifetime of the intermediate (τ) and its rotational period (*T*_R_).^63^ Maul and Gericke proposed use of the term “sequential” to describe the limiting case where the delay between the two bond-breaking events exceeds the average rotational period of the intermediate (τ/*T*_R_ > 1),^64^ resulting in a uniform angular distribution. Because the experimental angular distributions in the present work exhibit a decay component, this fragmentation channel represents an intermediate case, i.e., 0 < τ/*T*_R_ < 1, which we refer to as “asynchronous”.

**Figure 5:**
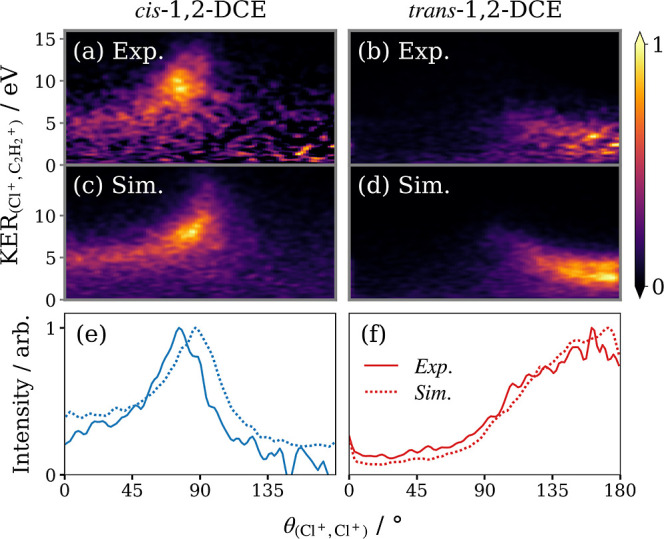
(a,b) Covariance maps from Figure 4c,d displayed in polar coordinates, where KER(Cl^+^,C_2_H_2_^+^) is the KER in the COM frame of the C_2_H_2_Cl^2+^ intermediate and  is the relative recoil angle between the Cl^+^ pair. (c,d) Simulated correlation maps for the asynchronous breakup of 1,2-DCE^3+^ into C_2_H_2_^+^ + 2Cl^+^. Each panel is normalized separately. Below the (solid) experimental and (dotted) simulated (Cl^+^, Cl^+^) relative recoil angle distributions are overlaid.

The final step is to transform into the center-of-mass (COM) frame of the C_2_H_2_Cl^2+^ intermediate,^6,35,63^ shown in the lower panels of Figure 4. This is possible only when working in terms of the fragment momenta, as opposed to their positions on the detector. The transformation is performed by subtracting the momentum that the reference Cl^+^ imparts to the plotted species. The momenta of Cl^+^ and C_2_H_2_^+^, which together constitute C_2_H_2_Cl^2+^, are equal and opposite in this frame. This distinctly enhances the twin arcs in each covariance map and provides an intuitive picture of the fragmentation dynamics. The initial dissociation step in the asynchronous breakup of the *cis*-1,2-DCE^3+^ induces counterclockwise rotation of C_2_H_2_Cl^2+^ in this frame, which pivots the Cl in the intermediate toward the recoiling Cl^+^ ion prior to secondary dissociation, thereby reducing the recoil angle between the Cl^+^ pair. The covariance maps in panels (c) and (d) of Figure 4 are replotted in Figure 5a,b, respectively, as a function of kinetic energy release (KER) in this frame versus the (Cl^+^, Cl^+^) relative recoil angle. The dynamics of the *cis* isomer breakup produces a distribution which decays from ∼80° (the angle for concerted breakup) toward 0°. A similar mechanism for *trans* acts to turn around the C_2_H_2_Cl^2+^ intermediate, giving a distribution which decays from 180° toward a smaller angle.

### Simulating Asynchronous Fragmentation Dynamics

To verify the assigned fragmentation mechanism for this channel, a second series of classical simulations of the three-body breakup of 1,2-DCE^3+^ were conducted, using a model modified to recreate asynchronous breakup dynamics. The alterations made to the base concerted Coulomb explosion model in large part follow the steps described in ref (6). Briefly, the simulation is executed as two separate Coulomb explosion events which model the primary and secondary fragmentation steps outlined in eq 3. The first step is propagated for the duration of the intermediate lifetime (*t*), which is determined by randomly sampling an exponential decay function with a characteristic lifetime τ. C_2_H_2_Cl^2+^ is then replaced by C_2_H_2_^+^ + Cl^+^, rotated through an angle
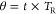
4

The separation of C_2_H_2_^+^ and Cl^+^ (*r*) is scaled to emulate extension of the C–Cl bond, modeled by an inverted exponential decay function
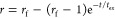
5

The asymptotic separation (*r*_f_) is determined by combining Coulomb’s law and the mean KER of the tail of the distribution in Figure 5a,b. The input parameters τ, *t*_ex_, and *T*_R_ are empirically chosen to reproduce the experimental results. For this reason, the simulations provide only qualitative insight into the nuclear dynamics of the intermediate dication.

Data simulated for the *cis* and *trans* isomers (using identical values of τ = 100 fs, *t*_ex_ = 100 fs, and *T*_R_ = 1 ps) are plotted in panels (c) and (d) of Figure 5, respectively. The simulation successfully reproduces the arcing features in the experimental covariance maps, supporting the interpretation of the rotational dynamics of the intermediate. The downward curvature toward smaller angles for the *cis* isomer is caused by the stretch of the C–Cl bond in the intermediate as a function of time and hence rotation of the intermediate. The upward curvature toward smaller angles for the *trans* isomer is produced by a combination of bond extension and rotation of the intermediate, both of which limit caging of the C_2_H_2_^+^ between the Cl^+^ pair, resulting in increased KER. The simulated angular distributions were found to be very sensitive to the ratio τ/*T*_R_. To achieve the resemblance to the experimental results seen in panels (e) and (f), it was necessary to tune this ratio to a small fraction, corroborating our statement that the dissociation is asynchronous.

In Figure S5 of the Supporting Information, the simulated data have been formatted into the same representation as the covariance maps seen in Figure 3, which displays the correlated momenta of all three fragment ions. Simulated correlation maps calculated using the 3D fragment momenta are also presented.

### Atomized Breakup

Fully mapping the correlated fragment momenta from a “complete” Coulomb explosion of 1,2-DCE into atomic ions would require the determination of a 6-fold correlation, which is beyond the capabilities of the current experiments. Neither is it feasible to examine such dissociation pathways using 3-fold covariance analysis, as the three-body breakup analysis, discussed in the previous section, relied upon using the momentum sum of the fragments to reduce the noise to a reasonable level. Consequently, one must rely on 2-fold covariance analysis and study the correlation between individual pairs of fragment ions. This section focuses on the correlated momentum of chlorine dications.

Through individual 2-fold covariance analyses, it can be confirmed that Cl^2+^ is not produced in correlation with any molecular ion fragments. A strong covariance signal is observed between Cl^2+^ and other atomic ions, including H^+^, C^+^, C^2+^, Cl^+^, and Cl^2+^, covariance maps for which are presented in Figures S6 and S7 of the Supporting Information. It is possible that Cl^2+^ is also produced in correlation with neutral atomic fragments, but it is considered very unlikely that Cl^2+^ is produced in coincidence with any molecular fragments. Hence, the production of Cl^2+^ can confidently be assigned to the dissociation of highly charged parent ions, which completely destroys the chemical bonding.

**Figure 6:**
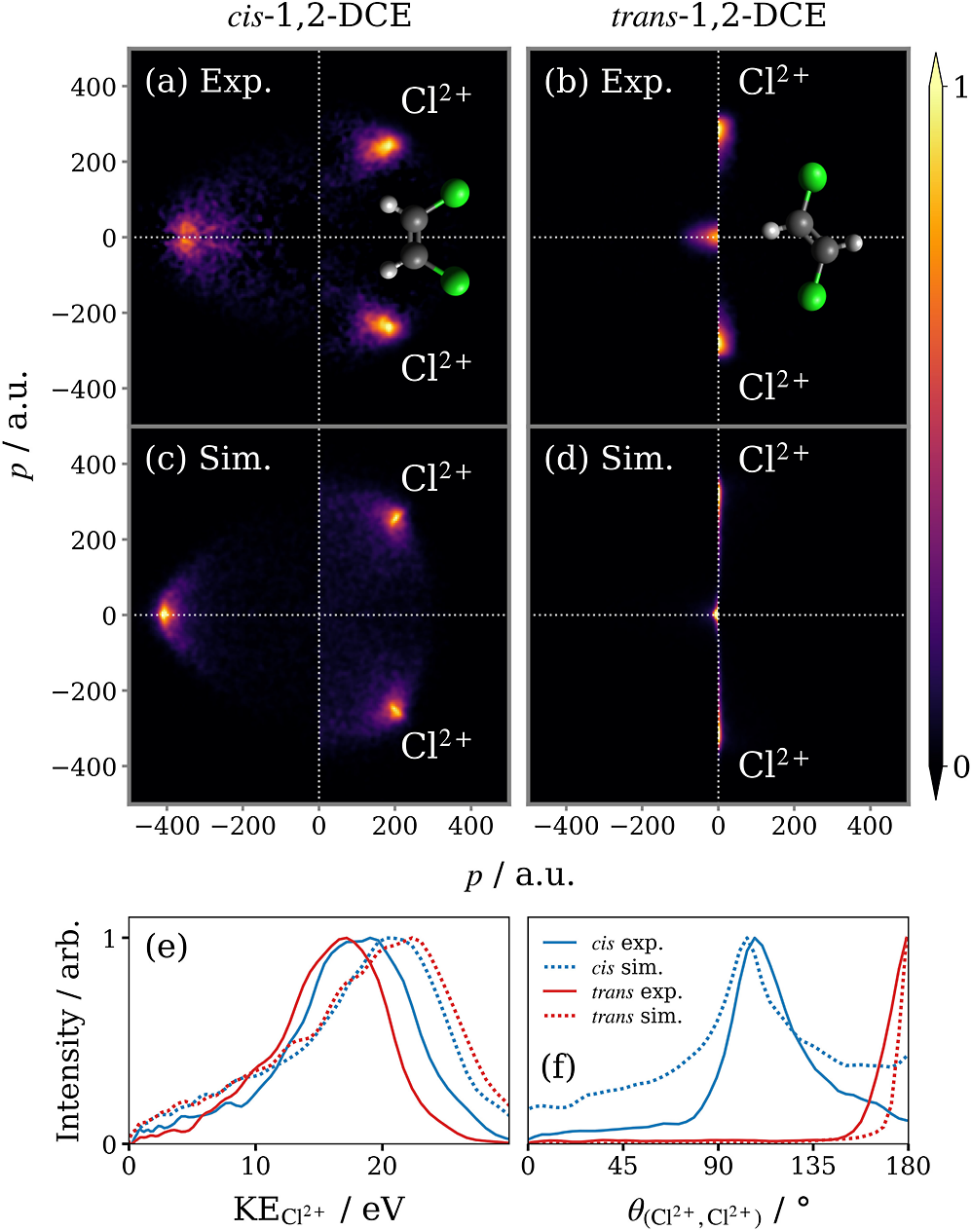
2D projected-momentum 2-fold covariance maps for the Coulomb explosion of (a) *cis*- and (b) *trans*-1,2-DCE which yields a pair of Cl^2+^ ions. The unlabeled feature in each covariance map corresponds to the sum of the momenta of all other fragments. Below (c,d) are the results of simulation of the Coulomb explosion of 1,2-DCE^8+^ into 2C^+^ + 2H^+^ + 2Cl^2+^. Each panel is normalized separately. In the bottom panels, (e) the Cl^2+^ KE and (f) (Cl^2+^, Cl^2+^) relative recoil angle distributions are overlaid.

The correlated momenta for a pair of chlorine dications, determined via 2-fold contingent covariance, are plotted in the top panels of Figure 6. Analogous to Figure 1b, the vertical axis is defined by the relative momentum of the two Cl^2+^ ions , such that the horizontal axis bisects the Cl^2+^ momenta. Once again, the relative recoil of the Cl^2+^ pair closely resembles the atomic positions of Cl in each isomer. Plotted in the left half of each covariance map is the residual momentum in this frame , which must equal the sum of the momenta of the other fragments (2C + 2H with undetermined charge). This feature contains indirect structural information that can be obscured in a different representation, such as the recoil frame. For example, the momentum sum of the missing fragments from the Coulomb explosion of *trans*-1,2-DCE is tightly distributed around the origin. This does not necessarily indicate that the fragments have near-zero momentum but rather that the pair of C fragments must recoil back-to-back, as must the H fragments, and hence that the molecule is symmetric about the line connecting the Cl atoms. This is confirmed by 2-fold covariance analysis between H^+^ + H^+^, and C^+/2+^ + C^+/2+^, also shown in Figure S7.

Panels (c) and (d) of Figure 6 show correlated fragment momentum maps for the many-body breakup of *cis*- and *trans*-1,2-DCE^8+^ into 2C^+^ + 2H^+^ + 2Cl^2+^, simulated using the basic Coulomb explosion model introduced in the last subsection of the Methods. This was the same fragmentation channel predicted for *Z* = 8 by the theoretical study of Zhou^41^ and was also the onset of the high *Z* regime in their study, where complete fragmentation into atomic ions was observed within 50 fs. The left-hand of the simulated plots displays the sum of the momenta of 2C^+^ + 2H^+^, rather than simply minus the momentum sum of the Cl^2+^ pair. The similarity between these simulated correlation maps and the experimental covariance maps in the panels directly above supports the claim that the 2-fold covariance analysis isolates signal from the complete dissociation of highly charged parent ions into many atomic ions, even though it only specifies the identities of two fragments.

Discrepancies can be attributed to the fact that, while the simulation emulates a single fragmentation channel, the experimental covariance maps likely contain contributions from several. For example, the intense features in (a) and (b) are notably more diffuse than simulation predicts. While the charge state of the Cl pair is specified, any combination of C or H with nonmatching charge states, i.e., C + C^+^, C^+^ + C^2+^, or H + H^+^, will break the symmetry of the charge distribution. Indeed, the work of Zhou predicts that the extra charge in the *Z* = 9/10 channels is accommodated by the C fragments. Contributions from such channels will broaden the relative recoil distribution of the Cl^2+^ pair.

We refrain from making any quantitative comparisons between the experimental and simulated Cl^2+^ KE distributions, as a model of point charges initiated from the equilibrium geometry of the molecule routinely overestimates the KE of fragments.^3,5,21^ The contribution of fragmentation pathways yielding neutral cofragments is one factor that could explain why the Cl^2+^ KE distributions are shifted down in energy relative to the simulated results, but this could also be the result of the slight extension of bonds on average during the ionization process.^5,6,11^ It is worth noting that the simulations qualitatively reproduce the similarity between the Cl^2+^ KE distributions for this pair of isomers.

### Comparison

Comparing the correlated momenta of the Cl^+^ pair from three-body breakup of 1,2-DCE^3+^, seen in Figure 3a,b, and the correlated momenta of the Cl^2+^ pair from atomized breakup of highly charged 1,2-DCE in Figure 6a,b, the features in the latter set of covariance maps are distinctly sharper, with no evidence of additional structure. This indicates that the Coulomb explosion of a highly charged parent ion yielding a Cl^2+^ pair is a concerted rather than an asynchronous process. Despite this difference in the nature of the fragmentation process, the average relative recoil angle of the Cl ion pair is similar for these two pathways (see Figures 3e,f vs 6f). For the *trans* isomer, both angular distributions peak at 180°. In the case of *cis*, the maximum intensity occurs at 111° for the Cl^+^ pair and 107° for the Cl^2+^ pair.

The breakup of 1,2-DCE^3+^ occurring via an asynchronous mechanism is to be expected given the relatively low charge state, *Z*. Similar dynamics have been observed for other small molecular trications.^6,35,63^ It is uncertain whether the theoretical method of Zhou^41^ predicts three body breakup as the outcome for the triply ionized *cis*-1,2-DCE as the simulation terminated at 50 fs, at which point only the C–C bond had broken. This certainly is not consistent with the formation of C_2_H_2_^+^, as observed experimentally. However, their work did predict C–Cl bonds being broken in a stepwise rather than a concerted manner for other low *Z* states.

As already noted, Zhou observed the onset of the high *Z* regime, where the theoretical method predicts direct and exclusive formation of atomic fragments, at *Z* = 8. For lower *Z,* the method predicts that polycationic molecular fragments, e.g., CCCl^3+^, remain intact when their simulations finished at 50 fs. No such fragments were observed in the current work. The study calculated the products for the dissociation of parent ions in their respective ground states, but it is likely that parent ions are prepared in a range of excited electronic states by the strong-field ionization process, which will exhibit varied dissociation dynamics.

For the purpose of using CEI to probe molecular structure on an ultrafast time scale, a distinction can be made between those channels in which the mapping between initial atomic positions in the parent molecule and final fragment momenta is straightforward and those which exhibit more complex mapping, which although often interesting in their own right, are not suitable for structural determination. The three body breakup of 1,2-DCE^3+^ represents an intermediate case. Although the bond breaking events can occur asynchronously, the dynamics of the intermediate during its short lifetime are not dramatic enough to completely obscure the structural information encoded in the fragment momenta. These dynamics lead to additional structure in the fragment momentum correlation map, which complicate interpretation, but the *cis* and *trans* isomers can still clearly be distinguished. The mapping is much more straightforward for the Coulomb explosion of more highly charged 1,2-DCE ions, particularly those producing pairs of Cl^2+^ ions, and can be understood using a model of point charges interacting under Coulomb’s law without additional modification. Any such channel is an obvious candidate for a future TR-CEI study on this pair of isomers, for instance, to investigate their interconversion through photoinduced *cis*–*trans* isomerization.

## Conclusions

2D projected-momentum covariance mapping has been introduced and demonstrated on 2D ion imaging data of the Coulomb explosion of the *cis* and *trans* isomers of 1,2-DCE. Working in terms of the 2D projected momenta of fragment ions greatly expands the array of available analysis tools. This study has included the application of a fragment momentum sum constraint in a 3-fold covariance calculation to isolate a three-body breakup channel and the use of various frames of reference which together allowed us to fully unravel the fragmentation dynamics in this channel. Though commonplace in 3D momentum imaging studies, such techniques are underutilized in analogous 2D studies and have been adapted here to achieve comparable results from a more limited 2D data set. These results exceed the level of detail typical of 2D ion imaging studies of molecular Coulomb explosion.

Two fragmentation pathways of 1,2-DCE polycations have been examined in detailed. The three-body breakup of triply charged parent ions into C_2_H_2_^+^ + 2Cl^+^ was identified as an asynchronous process. This was confirmed by a classical point charge simulation model of the dynamics. The many-body breakup of a highly charged parent ion which yields a Cl^2+^ ion pair, among other atomic ions, is a strictly concerted process. Both fragmentation pathways produce distinct signals for the *cis* and *trans* forms of the target molecule, allowing this pair of isomers to be unambiguously distinguished in a CEI experiment. Because it is a concerted process, the relationship between the initial atomic positions in the parent molecule and the relative momenta of the nascent fragments is more straightforward for the Coulomb explosion of a highly charged parent ion. This makes it the preferred choice of probe for a TR-CEI study which explores the photoinduced dynamics of this pair of species.

## Supporting Information Available

Discussion of the detector efficiency profile and possibility of clustering, ion count per laser shot distributions, contingent covariance demonstration, details on 3-fold covariance analysis, removal of impurity signal from covariance maps, description of the classical Coulomb explosion model, and additional 2-fold covariance maps (PDF)
