# Supplementary material for: Two-Dimensional Projected-Momentum Covariance Mapping for Coulomb Explosion Imaging
Source: J Phys Chem A. 2024 Apr 12;128(16):3220–9. doi: 10.1021/acs.jpca.4c01084 (PMC11056990; doi:10.1021/acs.jpca.4c01084)
Supplement: Supplementary file 1 — jp4c01084_si_001.pdf [file jp4c01084_si_001.pdf]

Supporting information for: Two-Dimensional Projected-Momentum Covariance Mapping For Coulomb Explosion Imaging

Joseph W. McManus,† Felix Allum,‡ Josh Featherstone,† Chow-shing Lam,† Mark Brouard†

† *Chemistry Research Laboratory, Department of Chemistry, University of Oxford, Oxford OX1 3TA, United Kingdom*

‡ *Current address: PULSE Institute, SLAC National Accelerator Laboratory, 2575 Sand Hill Road, Menlo Park, CA 94025, USA*

1 Defector efficiency profile

In the main text it is stated that the recorded yield of parent ions is very low because, under velocity-mapping conditions, ions with near-zero transverse momentum are focused to a point on the detector, producing overlapping flashes which saturate the detector. There is also the possibility that the detection efficiency of this central region is diminished relative to the rest of detector, because of accelerated degradation due to an increased density of ion impacts over time.

This is not expected to meaningfully affect the recorded distributions of the Coulomb explosion fragments of key interest. Only those ions which are ejected nearly parallel to the TOF axis would be detected with reduced efficiency. This is a very narrow segment of the full 3D distribution of ions (See Figure 1(b) of the main text, for example). This number was also decreased because the polarisation of laser pulses was parallel to the detector axis, and molecules with their most-polarisable electron-extraction axis parallel to the polarisation axis are preferentially ionised.

2 Confirming the absence of clustering

Two independent observations confirm that no parent molecule clusters were present in the molecular beam expansion for *cis* or *trans* isomers. First, no additional peaks are discernible in the TOF spectra (Figure 2 of the main text) beyond the range shown, and specifically, there is no intensity at the TOF predicted for the parent dimer, trimer, etc. Second, no high KE parent ions are observed in the velocity-map images of the parent ions. If parent dimer was present, for example, Coulomb explosion of the dimer dication would be expected to give rise to a pair of relatively high KE parent ions.

3 Contingent covariance

The ion count per laser shot distribution for each dataset is displayed in Figure S1. The boundaries of the bins used to divide up the data for contingent covariance analysis are shown by the dashed vertical lines. The bins were selected such that each contains the same number of laser shots.

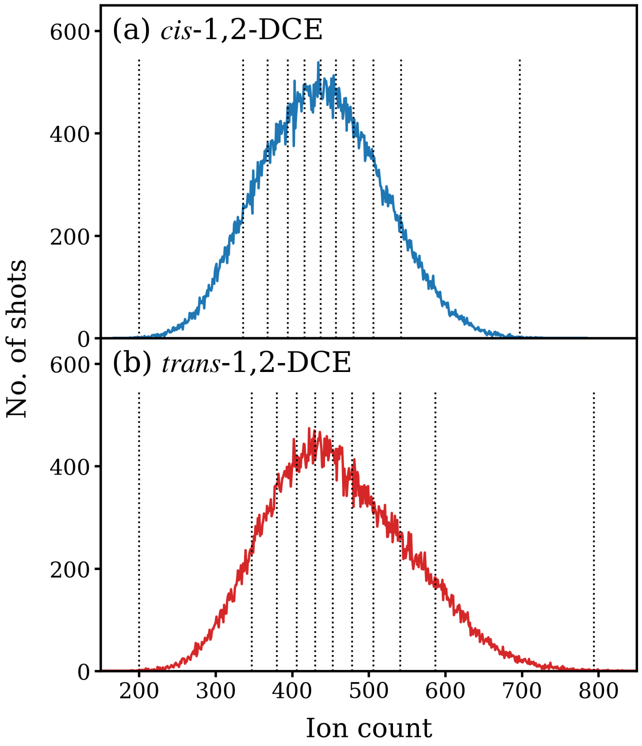

Figure S1: Ion count per laser shot distribution for (*top*) cis- and (*bottom*) trans-1,2-DCE. Vertical dotted lines indicate the boundaries of the bins used for contingent covariance calculations.

An example of the output of the contingent covariance analysis, side by side with the output of a standard covariance analysis for the same data, is shown in Figure S2. The contingent covariance calculation removes a substantial amount of false covariance, including entirely eliminating the signal at a (Cl^{2+}, Cl^{2+}) relative recoil angle below 45°. For the complete Coulomb explosion of *trans*-1,2-DCE, such a recoil angle is unphysical.

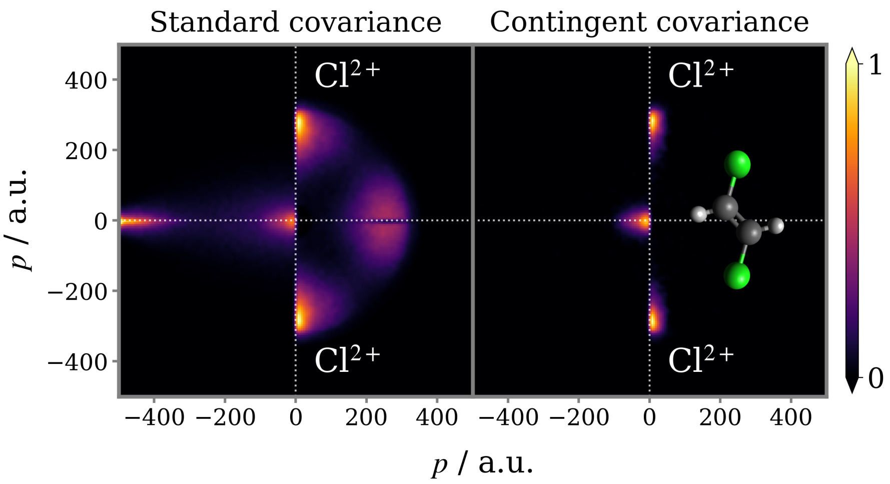

Figure S2: 2D projected-momentum two-fold covariance maps for the Coulomb explosion of *trans*-1,2-DCE which yields a pair of Cl^{2+} ions. The left panel has been calculated using a standard covariance analysis, and the right using a contingent covariance analysis, where the data was grouped into 10 subsets based upon ion count per laser shot. Each panel is normalised separately.

4 Three-fold covariance calculation

In the dataset for each isomer, the number of combinations of $C_2H_2^+$ + $2Cl^+$ with a projected momentum sum within the range ± 5 a.u. is a very small fraction of the total number. Using the fragment momentum sum as a filter prior to three-fold covariance analysis therefore reduced the number of combinations which needed to be considered by $\sim$99.9%. Due to very high average ion count per laser shot, this was a reduction from several billion combinations of $C_2H_2^+$ + $2Cl^+$ to several million over the $\sim$100,000 total accumulated laser shots for each target molecule. The number of combinations was therefore still on the order of tens per laser shot after filtering, so a covariance analysis remained necessary to suppress false correlation events.

This is best demonstrated by inspecting the maps for some of the individual terms which together equal the three-fold covariance:

$$\mathrm{cov}(A, B, C) = \langle ABC \rangle - \langle AB \rangle \langle C \rangle - \langle AC \rangle \langle B \rangle - \langle BC \rangle \langle A \rangle + 2\langle A \rangle \langle B \rangle \langle C \rangle. \quad (2)$$

Panels (a) and (b) of Figure S3 display two of the terms which contribute to the three-fold contingent covariance map for the three-body breakup of triply charged *cis*-1,2-DCE into $C_2H_2^+$ + $2Cl^+$, shown in (c). The first is the 'triple coincidence' term ($\langle ABC \rangle$) calculated from all combinations of $C_2H_2^+$ + $2Cl^+$ detected in the same laser shot. The second is the 'single coincidence' term ($\langle A \rangle \langle B \rangle \langle C \rangle$), which is instead calculated from all combinations of $C_2H_2^+$ + $2Cl^+$ across a random sample of laser shots, *i.e.* this map is constructed from exclusively false coincidence events.

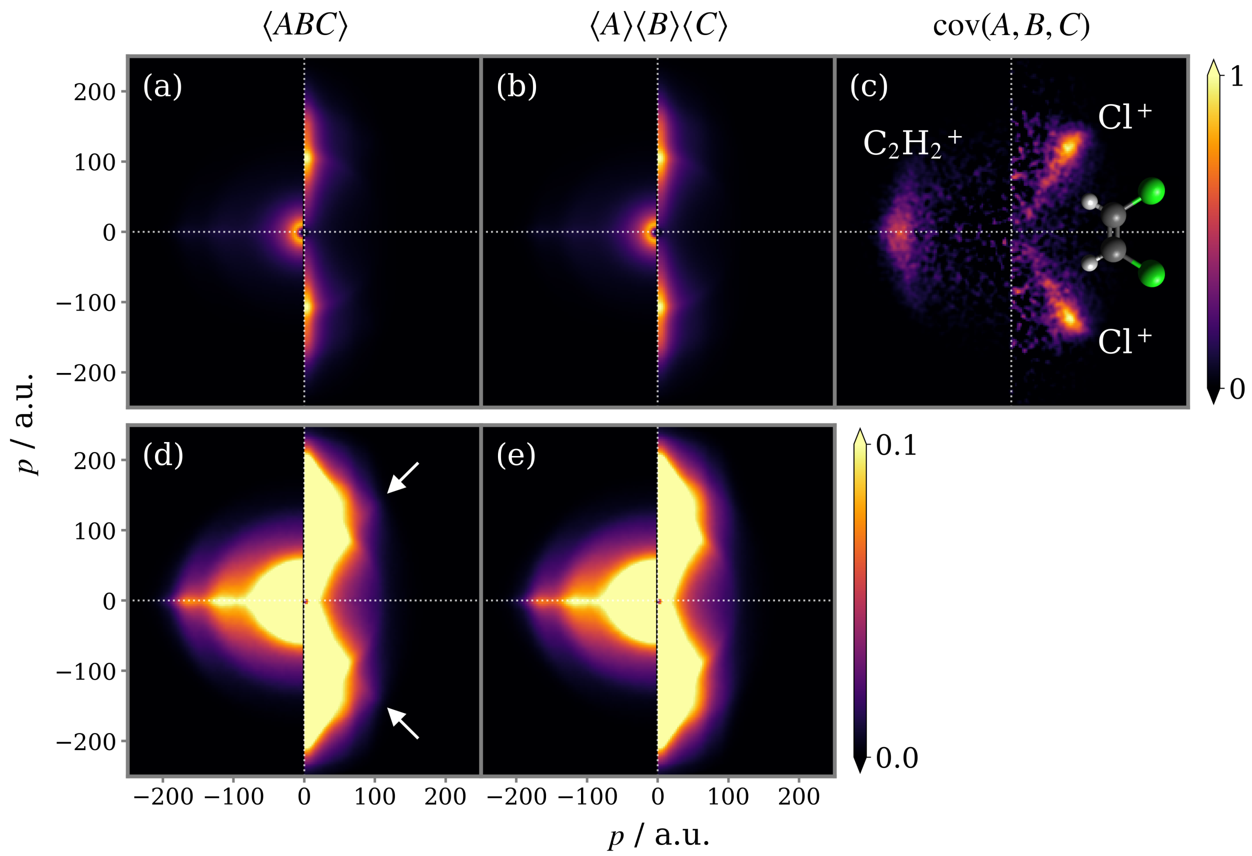

Figure S3: Calculated (a) triple coincidence term, (b) single coincidence term and (c) three-fold covariance for the combinations of $C_2H_2^+$ + $2Cl^+$ from *cis*-1,2-DCE with a fragment momentum sum within ±5 a.u. Each panel in the top row is normalised separately. The bottom panels display the triple and single coincidence terms on a saturated colour scale in order to highlights subtle differences between them, as indicated by the white arrows.

On a standard colour scale the triple and single coincidence terms are indistinguishable, and in fact so are all the other terms which are not shown here. This indicates that the triple coincidence map is dominated by false coincidence. The constraint on the fragment momentum sum alone is not sufficient to isolate the true coincidence events, and a coincidence analysis is indeed not appropriate. Only by saturating the colour scale to reveal the low intensity features can subtle differences be seen between the tripe and single coincidence terms, as shown in the lower panels of Figure S3. The white arrows highlight additional features in the triple coincidence map, which corresponds to the high momentum Cl^+ signal isolated in the covariance map.

5 Removing impurity signal from covariance maps

During data analysis it became apparent that the dataset for *cis*-1,2-DCE was, to a small extent, contaminated with signal arising from the *trans* isomer. This is observed when the correlated fragment momenta for the $C_2H_2^+$ + $2Cl^+$ fragmentation channel calculated

from the *trans* and *cis* datasets are compared, shown in panels (a) and (b) of Figure S4, respectively. Though (b) is dominated by the *cis* isomer signal, there is clearly a small component which resembles the features in (a). This is attributed not to the impurity of the original sample, but rather to insufficient cleaning between sample changes, as the dataset for *cis* was collected immediately following data acquisition for *trans*.

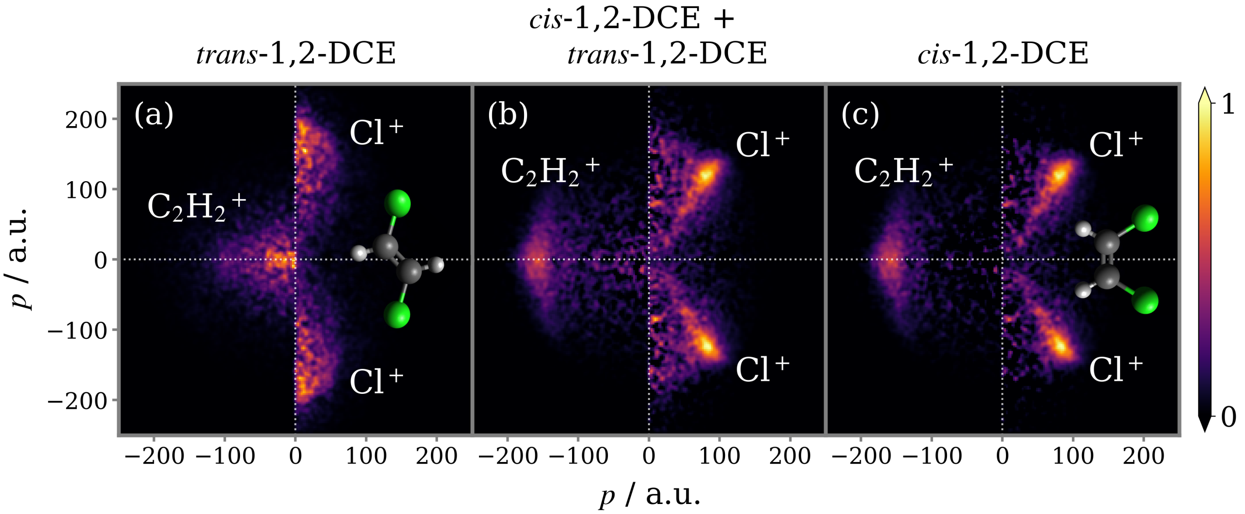

Figure S4: 2D projected-momentum three-fold covariance maps for the three-body breakup of 1,2-DCE^{3+} into $2Cl^+$ + $C_2H_2{}^+$, calculated from (a) the *trans* isomer dataset and (b) the *cis* isomer dataset - which is contaminated with signal from *trans*. Panel (c) displays the isolated *cis* signal, obtained by scaling (a) and subtracting from (b). Each panel is normalised separately.

To isolate the pure *cis* isomer signal, (a) is scaled and subtracted from (b), resulting in the covariance map seen in panel (c). The scaling was performed qualitatively to avoid over subtraction. This procedure was repeated for any covariance map calculated from the *cis* dataset in which the impurity signal was apparent.

6 Classical Coulomb explosion simulations

Here the basic Coulomb explosion model for concerted fragmentation is described in detail. The fragments are treated as point charges which interact exclusively under Coulomb's law. The charge on each fragment is located at its COM and is fixed, *i.e.* no charge redistribution. When the simulation is initiated the fragment trajectories are simply propagated until they have reached their tangential velocities. Figure S5 shows the correlation map for the Coulomb explosion of triply charged *trans*-1,2-DCE into $C_2H_2{}^+$ + $2Cl^+$, produced from 2D projected fragment momenta simulated by the model.

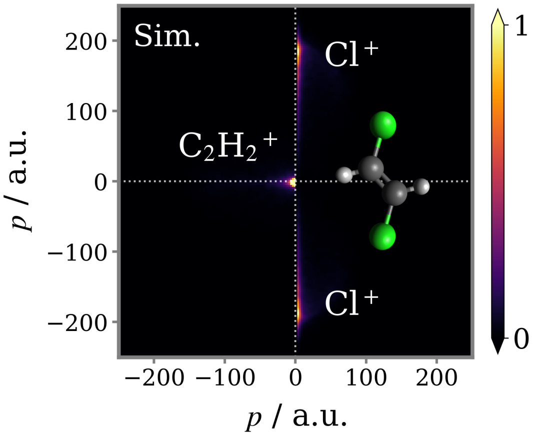

Figure S5: Simulated 2D projected-momentum correlation map for the three-body concerted dissociation of *trans*-1,2-DCE^{3+} into 2Cl$^+$ + $C_2H_2{}^+$.

For all simulations of the $C_2H_2{}^+$ + 2Cl$^+$ fragmentation channel, the structure of the $C_2H_2{}^+$ moiety is fixed to the same configuration as the equilibrium ground state geometry of the molecule, whilst the positions of the Cl atoms are sampled from a distribution which incorporates relevant vibrational modes. These include C–Cl symmetric/antisymmetric stretching, Cl–C–C–Cl in-plane scissoring/C–C–Cl bending and Cl–C–C–Cl out-of-plane twisting. For example, in each simulation the C–Cl bond length is selected from a Gaussian distribution, centred at the equilibrium geometry bond length, with a width of 0.1 Å. The molecule is randomly selected to be stretched either symmetrically, in which case both bonds are given the same length, or asymmetrically, in which case one bond is extended by the same amount the other is compressed.

The alterations made to provide a model of asynchronous fragmentation are outlined briefly in the main text, and described in detail in ref. 6. Here they are not expanded upon further. In Figure S6, the correlated momenta of $C_2H_2{}^+$ + 2Cl$^+$ from experiment and simulation are compared using the representation which displays the momenta of all three ions in the COM frame of the parent molecule. The asynchronous breakup model is able to reproduces features in the experimental covariance maps which the concerted breakup model could not, such as the tail in the Cl$^+$ momentum distribution for *cis*-1,2-DCE which trails from the point of maximum intensity down towards the origin.

For completeness, in the lower panels simulated correlation maps are shown which have been calculated using the 3D fragment momenta. In contrast to the 2D projected-momenta correlations map, the smearing of the features is eliminated in full 3D covariance, but otherwise their form is unchanged.

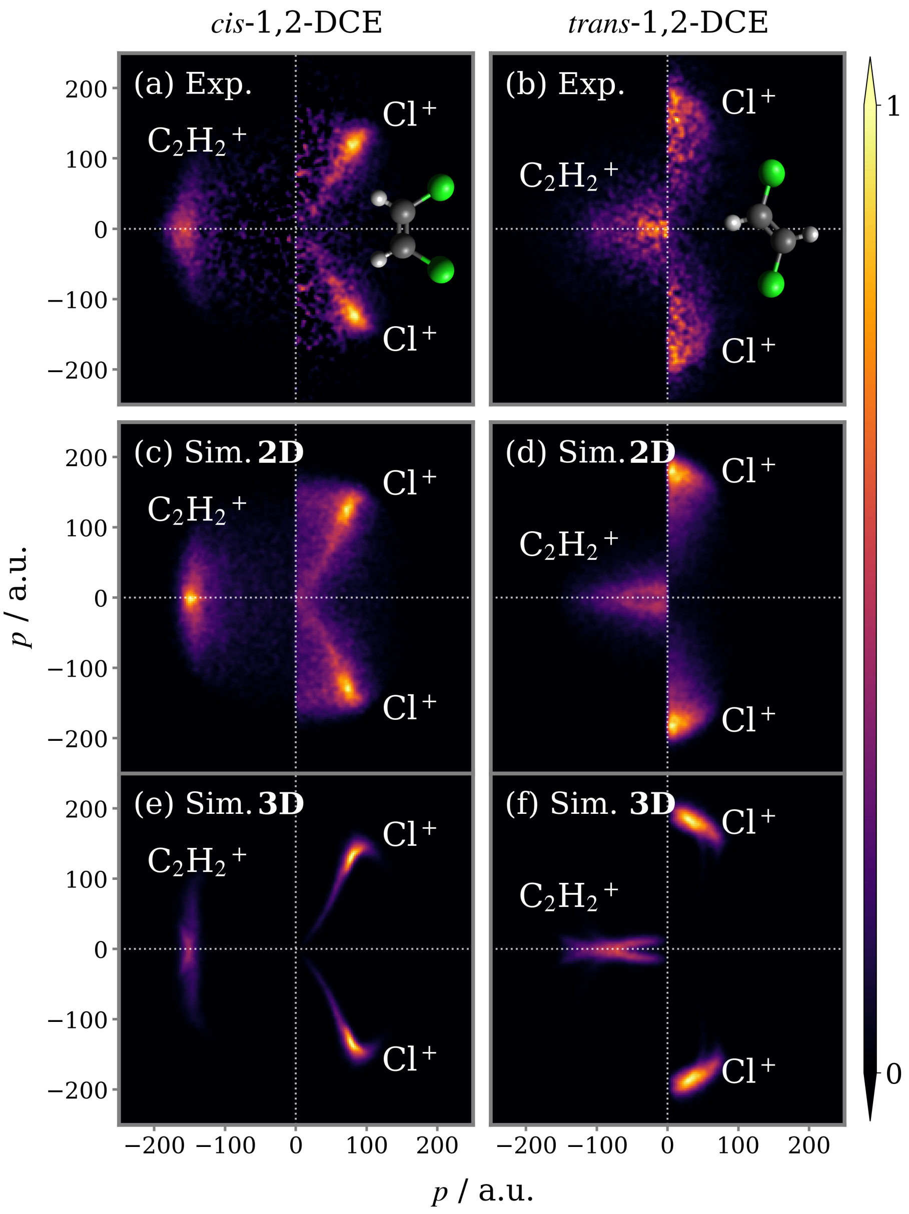

Figure S6: 2D projected-momentum three-fold covariance maps for the three-body breakup of (a) *cis*- and (b) *trans*-1,2-DCE^{3+} into 2Cl$^+$ + $C_2H_2{}^+$. Below are the results of simulation of this fragmentation channel using the asynchronous breakup model, calculated using (c, d) the 2D projected and (e, f) 3D fragment momenta.

7 Additional covariance maps

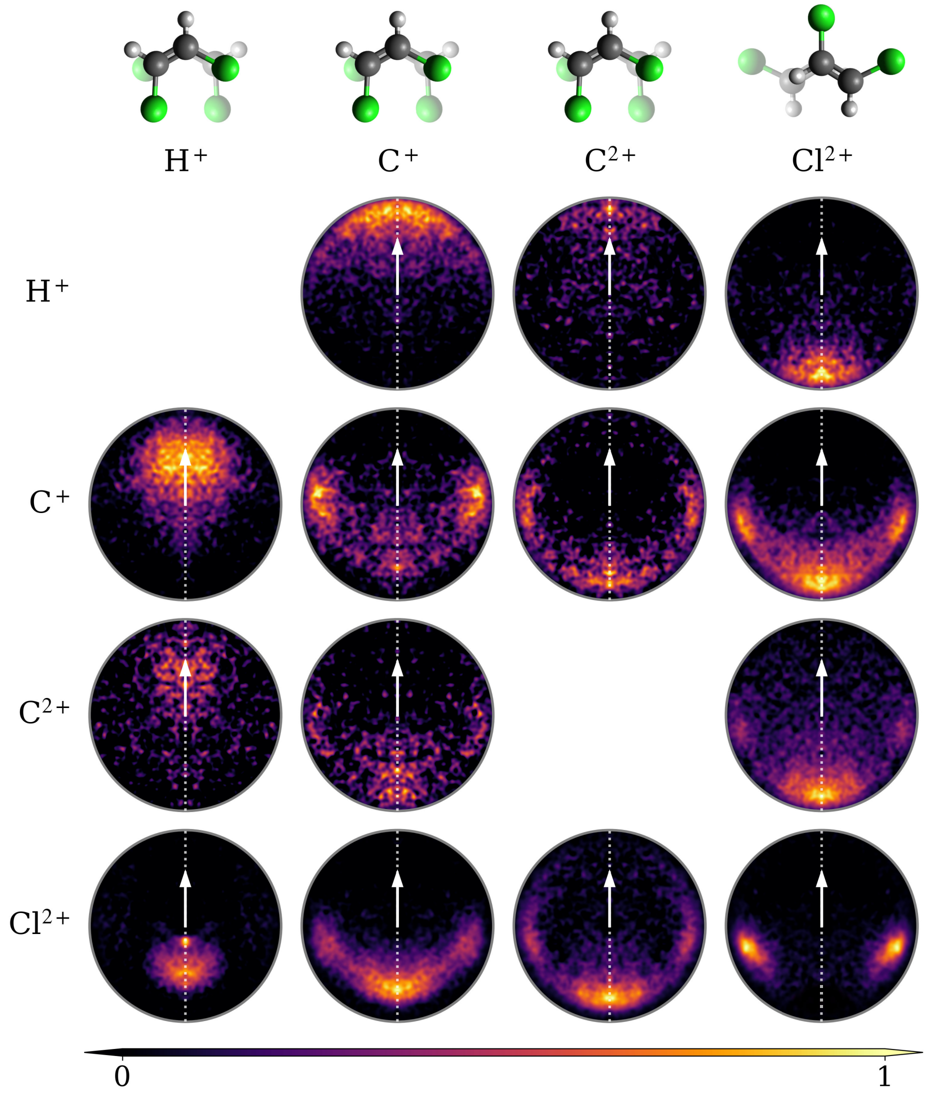

Figure S7: Recoil-frame two-fold covariance maps for the Coulomb explosion of *cis*-1,2-DCE. The reference and partner ion for each plot are labelled along the horizontal and vertical axes, respectively. The reference ion is constrained to the positive y-axis, as indicated by the arrow, and the positional distribution of the partner ion on the detector is plotted relative to this direction. Covariance maps with no statistically significant signal have been omitted.

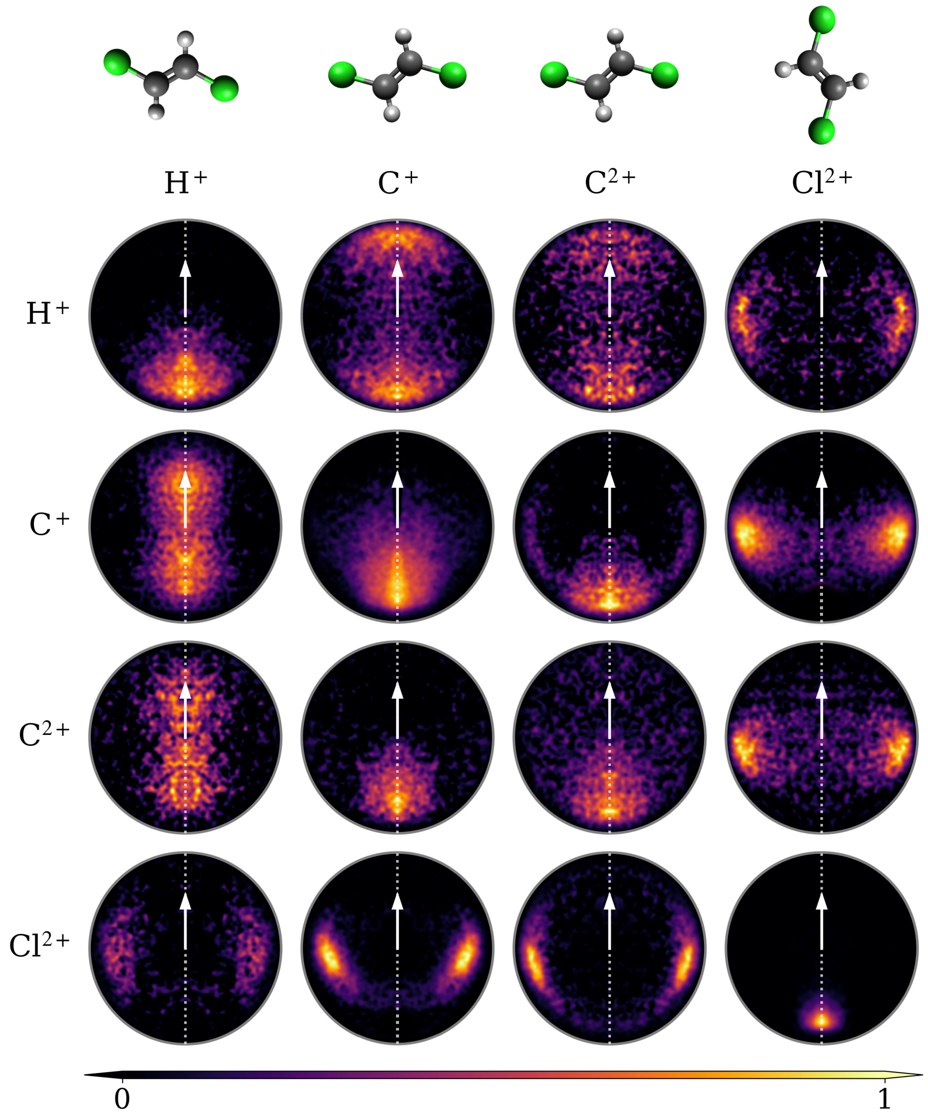

Figure S8: Recoil-frame two-fold covariance maps for the Coulomb explosion of *trans*-1,2-DCE. The reference and partner ion for each plot are labelled along the horizontal and vertical axes, respectively. The reference ion is constrained to the positive y-axis, as indicated by the arrow, and the positional distribution of the partner ion on the detector is plotted relative to this direction.
